# Prenatal repair of gastroschisis using partial carbon dioxide insufflation fetoscopy: lessons learned

**DOI:** 10.31744/einstein_journal/2023RC0543

**Published:** 2023-05-23

**Authors:** Gustavo Henrique de Oliveira, Gregório Lorenzo Acácio, Rodrigo Tadeu Russo Gonçalves, Javier Svetliza, Gustavo Yano Callado, Cristiane de Moraes Dias, Denise Cristina Mós Vaz-Oliani, Ramen H. Chmait, Denise Araújo Lapa

**Affiliations:** 1 Faculdade de Medicina de São José do Rio Preto São José do Rio Preto SP Brazil Faculdade de Medicina de São José do Rio Preto, São José do Rio Preto, SP, Brazil.; 2 Faculdade de Medicina de Taubaté Taubaté SP Brazil Faculdade de Medicina de Taubaté, Taubaté, SP, Brazil.; 3 Hospital do Servidor Público Estadual “Francisco Morato de Oliveira” São Paulo SP Brazil Hospital do Servidor Público Estadual “Francisco Morato de Oliveira”, São Paulo, SP, Brazil.; 4 Hospital Interzonal General de Agudos Dr. José Penna Bahía Blanca Argentina Hospital Interzonal General de Agudos Dr. José Penna, Bahía Blanca, Argentina.; 5 Faculdade Israelita de Ciências da Saúde Albert Einstein Hospital Israelita Albert Einstein São Paulo SP Brazil Faculdade Israelita de Ciências da Saúde Albert Einstein, Hospital Israelita Albert Einstein, São Paulo, SP, Brazil.; 6 Keck School of Medicine University of Southern California Los Angeles CA United States Division of Maternal-Fetal Medicine, Keck School of Medicine, University of Southern California, Los Angeles, CA, United States.; 7 Hospital Israelita Albert Einstein São Paulo SP Brazil Hospital Israelita Albert Einstein, São Paulo, SP, Brazil.

**Keywords:** Gastroschisis, Abdominal wall, Fetoscopy, Fetal therapies, Fetus, Infant, newborn, Carbon dioxide, Insufflation, Fetal distress

## Abstract

We report the long-term outcomes of a case of prenatal gastroschisis repair using a fully percutaneous fetoscopic approach with partial carbon dioxide insufflation. Surgery was performed as an experimental procedure before the scheduled elective birth. The fetal intestines were successfully returned to the abdominal cavity without any fetal or maternal complications. Ultrasonography performed 24 hours later revealed bowel peristalsis and no signs of fetal distress. After 48 hours, partial extrusion of the small bowel was observed, and the fetus was delivered. Gastroschisis repair was immediately performed upon delivery using the EXIT-like procedure as per our institutional protocol. The newborn did not require assisted mechanical ventilation, was discharged at 14 days of age and was then exclusively breastfed. At 3-year follow-up, the patient had no associated gastroschisis-related complications. This is the first case of prenatal repair of gastroschisis, which provides baseline knowledge for future researchers on the potential hurdles and management of prenatal repair.

## INTRODUCTION

Gastroschisis (GS) occurs due to an abdominal wall defect through which the abdominal viscera herniate without the protection of the peritoneum, leaving the fetal bowel floating freely in the amniotic fluid during pregnancy.^( 1 )^ Surgical correction of the abdominal defect is routinely performed shortly after birth.^( 1 )^ Although surgical correction is relatively simple, the vast majority of newborns require ventilatory support and parenteral nutrition, and some are subjected to extensive bowel resection due to necrosis or atresia.^( 2 - 4 )^ Surgical correction can be accomplished by primary defect closure, but may require the placement of a silo.^( 1 )^ Although mortality is considered low in tertiary centers, it is not uniform worldwide and can reach up to 30% in Brazil.^( 5 )^ There is also high morbidity owing to the time it takes for the bowels to resume peristalsis and assume normal absorption capacity. When primary repair cannot be accomplished, a silo is placed to allow the progressive return of the intestines to the abdominal cavity. This may be placed for days, increasing the risk for infection and death.^( 2 , 6 )^

Bowel dysfunction is mainly attributed to ischemia of the herniated loops that slowly enlarge while the size of the abdominal wall defect remains the same, thereby causing constriction. Furthermore, inflammation of the intestines can occur due to direct contact of the intestinal serosa with the amniotic fluid.^( 7 , 8 )^ Amnioexchange was proposed to mitigate this damage and was tested in a prospective randomized trial in humans, but this intervention did not improve fetal outcomes.^( 7 )^

Approximately 15% of fetuses with gastroschisis can be classified as ‘complex’, even in the prenatal period.^( 9 - 11 )^ Complex gastroschisis is defined as gastroschisis accompanied by intestinal atresia, necrosis, perforation, volvulus, or necrotizing enterocolitis; whereas simple gastroschisis does not involve any of these conditions. As shown in a recent meta-analysis, the morbidity and mortality associated with gastroschisis are generally clustered within these ‘complex’ cases compared to the simple cases, including in-hospital mortality (16.7% *versus* 2.2%), short bowel syndrome (27.0% *versus* 1.7%), necrotizing enterocolitis (14.7% *versus* 8.0%), length of stay (90-130 days *versus* 23-44 days), and time required for oral feeding (90-178 days *versus* 30-36 days), respectively.

Even non-complex cases can have severe complications^( 2 , 9 , 11 , 12 )^ including abdominal compartment syndrome, intestinal stenosis, necrosis, and extensive intestinal resections that ultimately lead to short intestines and other malabsorption syndromes.^( 13 , 14 )^ Long-term outcomes of non-complex cases include abdominal pain and the need for additional surgery,^( 14 )^ and overall mortality rates can reach 9-25% depending on the center.^( 2 , 15 - 17 )^ Furthermore, recent evidence has shown that survivors may have subtle abnormal neurodevelopment.^( 14 , 18 , 19 )^ Previous studies have found that children with gastroschisis may be particularly at risk for executive functioning difficulties despite an intelligence quotient (IQ) that is within normal limits.^( 11 , 19 )^ Visual impairment and sensorineural hearing loss have been reported as well. Although the causes of this delay are not yet known, it can be hypothesized that nutritional challenges, repeated general anesthesia, repeated surgical interventions, and prolonged hospitalization may play a role. This is why treatment before birth, which has been studied for over a decade in animal models^( 9 )^ may become a viable alternative with the potential to reduce the length of stay, morbidity, and mortality.

Fetal correction could eliminate the need for postnatal surgery by potentially treating the defect in utero. This approach could permit the reestablishment of intestinal transit before birth and enables immediate mother and child contact. Owing to the need for immediate surgery, the lack of maternal/neonatal contact may also contribute to behavioral disorders.^( 14 )^

However, the risks of fetal surgery in mothers cannot be overlooked, and these aspects have long been a topic of debate.^( 12 , 13 )^ Recent technical improvements in fetoscopy have increased maternal safety, and the use of partial CO_2_ insufflation in the uterus has allowed for more complex yet minimally invasive fetal interventions. Moreover, concerns about the safety of CO_2_ use in uterine insufflation raised in the past decades have been mitigated, pushing the boundaries of fetal surgery.^( 20 , 21 )^

However, one technique, the zero-time correction technique called EXIT-like,^( 22 )^ showed some promising results, including improved neonatal outcomes, such as time to discharge and oral feeding.

Our unit adopted the EXIT-like technique in 2014 under the guidance of Dr. Javier Svetliza,^( 22 )^ which has been the preferred method for postnatal treatment of gastroschisis. Briefly, a planned delivery is scheduled at 36-37 weeks and the herniated viscera are returned to the abdominal cavity before the baby starts to breathe, while still connected to the maternal circulation.

Our team has pioneered the prenatal repair of spina bifida using CO_2_ insufflation for fetoscopy.^( 23 )^ We used a completely percutaneous technique and have already performed over 100 cases of fetoscopy to correct open spina bifida with low morbidity and no maternal mortality.^( 24 )^

To the best of our knowledge, this is the first reported case of gastroschisis treated before birth in the literature.

## CASE REPORT

A primigravida was referred to *Hospital da Criança e Maternidade* (HCM) of the *Faculdade de Medicina de São José do Rio Preto* , which is one of the national reference center for the treatment of gastroschisis, due to a finding of gastroschisis in the fetus during prenatal care. The patient’s delivery was scheduled at 35 weeks of pregnancy using an EXIT-like surgical approach, as per our institutional protocol.

In this scenario, we offered fetoscopy as an experimental procedure, given our team’s high level of expertise in using CO_2_ insufflation and the assumed low maternal risk.

By timing the prenatal repair close to the gestational age of the anticipated scheduled cesarean delivery according to our institutional protocol and proceeding with EXIT-like repair in case of any maternal or fetal complications, we assessed the risks associated with the fetal procedure as relatively low. If successful, we could prevent neonatal anesthesia and surgery, favoring improved neonatal outcomes, including breastfeeding and mother-child interactions.

A fully percutaneous fetoscopic approach was discussed and agreed upon by the fetal medicine, obstetrics, neonatology, and pediatric surgery teams. The day before surgery, we rehearsed the planned repair in an animal model with all the teams present.

**Figure 1:**
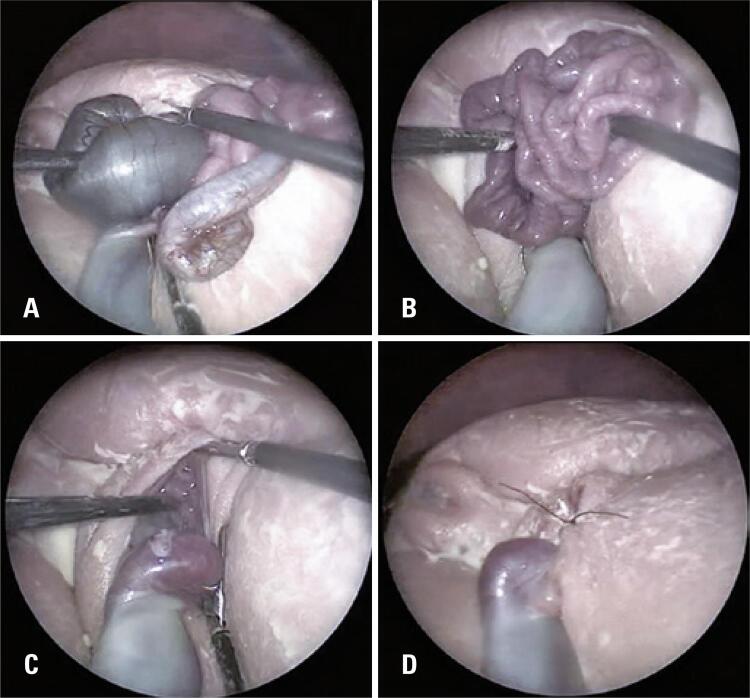
Fetoscopic images during the gastroschisis case repair. (A to C) Note the complete reduction of the herniated viscera and (D) the stitch used to suture the abdominal wall defect

Fetoscopy was performed at 33 weeks and 5 days of gestation. Surgery was performed under total intravenous (IV) anesthesia with propofol (2 or 3μg/mL, IV targeted controlled infusion). The induction of anesthesia was performed with fentanyl (5μg/kg, IV) and rocuronium (0.6mg/kg, IV), followed by remifentanyl (0.2μg/kg/min). Total IV anesthesia was adjusted for the depth of anesthesia using a bispectral index. The percutaneous Seldinger technique was then used to access the uterine cavity for the placement of four trocars: three 11-French vascular introducers (Terumo^®^, Tokyo, Japan) and a 5mm balloon-tipped laparoscopic trocar (Applied Medical, Rancho Santa Margarita, CA, USA). Subsequently, almost all the amniotic fluid was removed, and heated (35^o^C); and humidified (95% relative humidity) CO_2_ was insufflated into the uterus at a flow of 30L/min and a maximum pressure of 12±2mmHg. Camera and surgical graspers for intestinal manipulation were introduced through the trocars and, after grasping the edge of the gastroschisis orifice, the bowel loops were returned to the abdominal cavity using 3.5mm bowel laparoscopic graspers smoothly and without damage. First, the more dilated colonic loop was replaced followed by the less dilated small bowel loops, until the gastroschisis correction was completed ( Figure 1 ). A single “X” stitch suture with synthetic mononylon 4.0 was used to close the paraumbilical opening, aiming not to cause excessive constriction to the umbilical cord insertion. The entire procedure took 102 minutes, and no maternal or fetal complications were observed. We prepared for an emergency C-section and had an operating room available to operate on the neonate for a possible EXIT-like procedure.

The video of the fetoscopic surgery can be watched using the link: https://youtu.be/rJD1z-6gUgA.

On the first postoperative day, ultrasonography showed no viscera outside the abdominal cavity, and bowel peristalsis was noted. Continuous fetal heart rate monitoring was performed for 48 hours after surgery and reassuring fetal heart rate patterns were observed. Doppler velocimetry of the umbilical and middle cerebral arteries was normal and fetal movements were observed. However, the ductus venosus showed an increase in pulsatility index with a reverse a-wave, as well as pulsatility of the intrahepatic umbilical vein. Since no other signs of fetal distress were observed, an expectant approach was chosen. We used lower-extremity Doppler waveforms to identify indirect signs of compartment syndrome. The femoral arteries showed no changes in pre-surgical values or waveforms.

**Figure 2:**
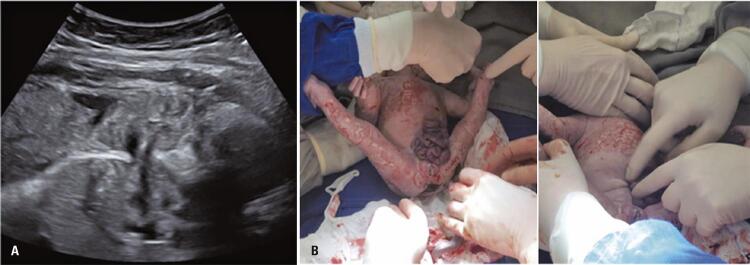
(A) Ultrasound image on second postoperative day revealed externalized small bowel loops between the umbilical cord and the suture point; (B) The portion of small intestinal loops after birth but before the gastroschisis repair using the EXIT-like procedure

**Figure 3:**
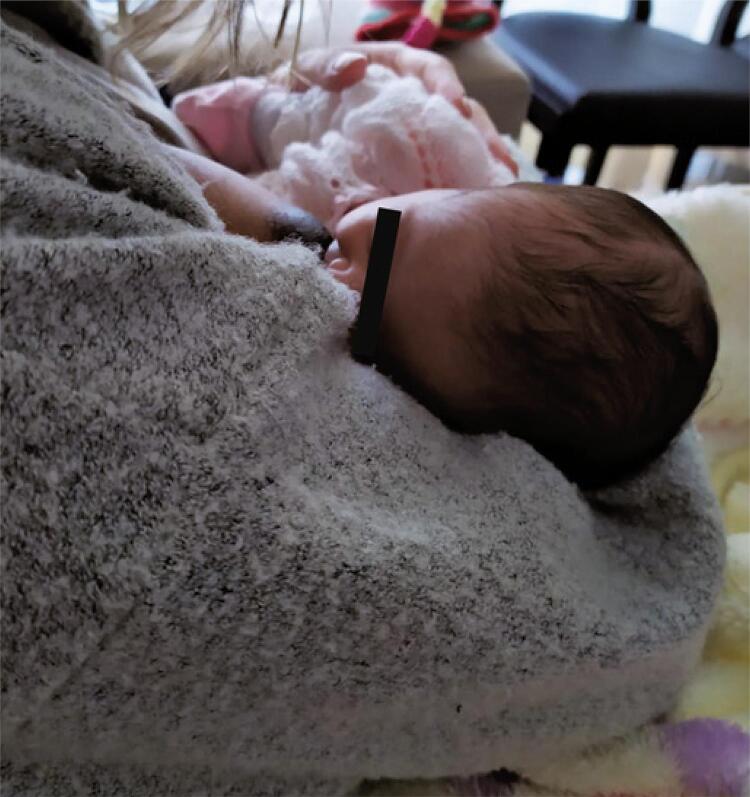
The newborn baby at home breastfeeding 15 days after delivery

On the second postoperative day, we noted the presence of small bowel loops externalized through an approximately 3mm opening on ultrasonography, located between the abdominal wall suture and the insertion of the umbilical cord ( Figure 2A ). Only a part of the small intestine was herniated, and no segment of the colon was observed outside the abdominal wall ( Figure 2B ). We decided to deliver the baby and successfully performed an EXIT-like repair. The procedure was accomplished without technical difficulty, and no ischemia of the exteriorized intestine was observed. The female newborn weighed 1,980g and had APGAR scores of 9 and 10. No ventilatory support was necessary. After seven days of exclusive parenteral nutrition, four days of combined parenteral/enteral nutrition, and four days of exclusive enteral nutrition, the newborn was discharged on the 14^th^ day of life and began breastfeeding ( Figure 3 ). Currently, the infant is 46 months old and has had an uneventful outcome without complications such as bowel obstruction, short intestine, bowel surgery, or pain. The mother was also healthy and had no complications.

The study was approved by the Research Ethics of *Faculdade de Medicina de São José do Rio Preto* under CAAE: 69013123.1.0000.5415; #6.059.172, as an experimental procedure, and informed consent from the patient was obtained.

## DISCUSSION

Fetal surgery has evolved considerably, and maternal safety has greatly increased with the emergence of minimally invasive fetoscopic techniques. In percutaneous fetoscopy, ultrasound-guided percutaneous punctures are used instead of laparotomy and hysterotomy required for open surgery, reducing the maternal morbidity associated with laparotomy and reducing the risk for uterine rupture to nearly zero.^( 24 , 25 )^ Unlike in open surgery,^( 26 )^ the mother may still have an option for vaginal delivery in the present and subsequent pregnancies with percutaneous fetoscopy.

The particularities of our protocol, such as scheduled late preterm delivery and the awareness that our group was planning to start a Phase I trial, led us to offer the procedure in this case. We balanced the risks and benefits to the patient and her baby prior to the procedure.^( 25 )^ The patient was informed of the novel aspects of this case and its potential risks and benefits, and informed consent was obtained.

When we offered this procedure in 2019, the concept of potential selection criteria had not yet been established.^( 9 )^ Since then, criteria have been developed to classify patients into simple *versus* complex gastroschisis.^( 27 )^

Several valuable lessons can be learned from this case study. It became apparent that in utero, replacement of the bowel with the fetus may increase intraabdominal pressure. This has two important consequences. First, the increased intraabdominal pressure may have caused extrusion of the viscera from the abdomen. Second, increased intraabdominal pressure may lead to compression of the abdominal umbilical vein, causing secondary fetal venous Doppler abnormalities. Although the fetal status remained reassuring in our case, the abnormal ductus venosus waveform was worrisome, and the long-term effects could not be assessed as delivery occurred shortly afterwards.

The present case demonstrates that the reintroduction of fetal bowel loops into the abdominal cavity is a simple, safe, and probably reproducible procedure. The Achilles heel seems to be related to the repair of the abdominal wall without constricting blood flow through the umbilical vessels and avoiding fetal compartment syndrome. The observed change in the pattern of the ductus venous, even in the absence of acute fetal distress, may indicate that an increase in intraabdominal pressure upon the return of the viscera can cause circulatory changes. In an animal model, fetal death was frequently observed after correction and was mainly attributed to obstruction of blood flow at the level of the umbilical cord insertion site.^( 28 )^ This may be the main challenge to overcome in future cases. In our case, we observed repeat herniation of the small bowel loops through a very small opening located just between the suture point and the insertion of the umbilical cord. As delivery occurred 48 hours after fetoscopy, it was not possible to analyze whether the change in the venous duct would be transient or permanent and if this would lead to fetal distress. We believe that an increase in abdominal pressure may explain the occurrence of herniation through such a narrow area.

Therefore, an important hurdle for prenatal repair will be the degree of tension in the closure of the paraumbilical defect. We believe that a tight closure is important to prevent the recurrence of bowel herniation, as seen in the present case, but that it must be weighed against the risk of constriction of the umbilical vessels at the insertion site of the cord. Further studies should determine the optimal surgical approach for prenatal closure of the defect.

## CONCLUSION

To our knowledge, this is the first case report describing an in-utero fetoscopic repair of gastroschisis. Our case demonstrates that prenatal gastroschisis treatment is feasible and may improve neonatal outcomes. However, the risks of constriction of the umbilical cord in the area of repair still needs to be addressed. Further feasibility and safety studies are necessary before prenatal gastroschisis can be recommended as a standard procedure.
